# Schizoaffective disorder: Nosological controversies and absence of specific treatment guidelines

**DOI:** 10.1192/j.eurpsy.2021.1441

**Published:** 2021-08-13

**Authors:** M. Valtueña García, R. Combina Fescina, A. Vázquez González, C. Ludwig, A. González Suarez, J. López Fernández, C. Huergo Lora, S. Ocio León, L. Lago García

**Affiliations:** 1 Psiquiatría, Hospital Vital Álvarez Buylla, Mieres, Asturias, Spain; 2 Psiquiatría, Área VIII, Asturias, Spain; 3 Primary Care, Área VIII, Asturias, Spain; 4 Psiquiatría, Hospital Vital Álvarez Buylla, Asturias, Spain

**Keywords:** schizoaffective disorder, Paliperidone Long Acting Injection (PLAI), multidisciplinary treatment plan, nosological controversies

## Abstract

**Introduction:**

Schizoaffective disorder is a psychotic disorder of controversial nosological entity. Affective symptomatology and psychotic features of varying intensity coexist simultaneously in him throughout evolution. The lack of consensus on the existence of this entity determines its diagnostic delay and the absence of specific treatment guidelines.

**Objectives:**

To review the diagnostic criteria for schizoaffective disorder and the published scientific evidence on the efficacy and safety of the different therapeutic options available. To analyze the efficacy of a multidisciplinary treatment plan implemented in an intensive follow-up program, presenting the evolution of a clinical case.

**Methods:**

To review the psychiatric history and psychopathological evolution of a patient diagnosed with schizoaffective disorder from the beginning of an intensive follow-up program in a day center to the present. Review the existing scientific evidence on the usefulness of the treatments used in this nosological entity.

**Results:**

This is a longitudinal and retrospective study of a clinical case in which the areas for improvement are analyzed before implementing a multidisciplinary therapeutic program and the favorable results obtained today. Currently, the patient is euthymic and attenuated and chronic positive and negative symptoms persist that do not interfere with his functionality.

**Conclusions:**

From the implementation of an individualized, personalized and multidisciplinary maintenance treatment plan, an overall improvement in psychopathological stability and functional recovery is observed. Among the psychopharmacological options in this patient, Paliperidone Long Acting Injection (PLAI) stands out for its long-term efficacy and safety.

